# Mass spectrometry-based intraoperative tumor diagnostics

**DOI:** 10.4155/fsoa-2018-0087

**Published:** 2019-03-07

**Authors:** Lorena Hänel, Marcel Kwiatkowski, Laura Heikaus, Hartmut Schlüter

**Affiliations:** 1 Institute of Clinical Chemistry and Laboratory Medicine, University Medical Center Hamburg-Eppendorf, Martinistr. 52, 20246 Hamburg, Germany; 2 Small Animal Clinic, University of Veterinary Medicine Hannover, Bünteweg 9, 30559 Hannover, Germany; 3 Department of Pharmacokinetics, Toxicology and Targeting, University of Groningen, Antonius Deusinglaan 1, 9713 AV Groningen, The Netherlands

**Keywords:** cancer, DESI, iKnife, intraoperative tumor diagnostics, MasSpec pen, PIRL, real-time diagnosis, surgery, tumor margin

## Abstract

In surgical oncology, decisions regarding the amount of tissue to be removed can have important consequences: the decision between preserving sufficient healthy tissue and eliminating all tumor cells is one to be made intraoperatively. This review discusses the latest technical innovations for a more accurate tumor margin localization based on mass spectrometry. Highlighting the latest mass spectrometric inventions, real-time diagnosis seems to be within reach; focusing on the intelligent knife, desorption electrospray ionization, picosecond infrared laser and MasSpec pen, the current technical status is evaluated critically concerning its scientific and medical practice.

## Lay abstract

The correct assessment of tumor margins is a key challenge during surgical excision. Latest developments in research strive for the invention of a method to detect the tumor spread intraoperatively, enhancing the therapeutic outcome. Techniques coupling potential or established surgical instruments to mass spectrometric analysis are of great importance in the upcoming market. Methods using electrospray (desorption electrospray ionization), an electrocautery knife (intelligent knife), a surgical laser (picosecond infrared laser) and a drop of water (MasSpecPen) applied on the tissue surface are discussed in this review as new sampling methods.

## The establishment of mass spectrometry-based approaches for intraoperative diagnostics

The most common treatment for cancer is the complete surgical removal of the tumor in combination with additional therapeutic steps such as chemotherapy and radiation. Usually, for complete solid tumor resection, the surgeon has to exactly locate the tumor tissue and evaluate its extent based on macroscopic criteria, imaging techniques and background knowledge about the type of malignancy's behavior [1–3]. Following the principle of tissue-conserving surgery [4], the physician is confronted with the narrow window between resecting too much healthy tissue and not removing all tumor cells. The more precisely the extent of the tumor can be located, the better the surgeon can circumscribe the excision margins. Even though tissue of affected fields might be histologically unsuspicious, after tumor resection there is an increased risk of tumor recurrence [5].

These circumstances led to the need for improved intraoperative tumor diagnostics tools. In this review, techniques based on mass spectrometric approaches such as the intelligent knife (iKnife), desorption electrospray ionization (DESI), picosecond infrared laser (PIRL) and the MasSpec pen are critically evaluated regarding their potential to detect the molecular phenotypes of typical biomolecule cancer profiles. Universally, we generated the term ‘intelligent scalpel’ to describe techniques combining a surgical cutting device on-line with mass spectrometry (MS), equipped with appropriate algorithms for signal processing and interpretation.

## Tumor margin behavior

To excise a tumor in its complete volume, understanding of behavior during its growth is essential. Tumor tissue is embedded in a ‘transition zone’; cancerous and noncancerous cells are located in the vicinity, complicating the differentiation between healthy and tumorous tissue macroscopically, especially in early nonvisible tumor stages, named carcinoma *in situ* [6].

Broadly, the described transition zones consist of cells in a precancerous state with the same morphology, but different biochemical properties and therefore functional variabilities. According to the ‘field cancerization theory’, long-term injuries can promote the growth of carcinogenic alterations [5]. Within this preneoplastic field, histological changes may occur, but these changes are not always detectable, and therefore undetected tumor cells may remain in the operation field [7]. For instance, in the case of breast cancer with an invasive carcinoma or a carcinoma in situ, around 20% of the patients in a three year period need a reoperation to remove the tumor grown from positive margins, which were not excised during a first breast-conserving surgery [8]. In cancer forms like head and neck cancer [9–11], gastrointestinal cancer [12], colorectal cancer [13], skin cancer [14] and Barrett's esophagus [15], field cancerization has been described. Through the stages of field cancerization, molecular lesions such as genetic mutations and epigenetic changes can be detected, leading the cells to progress into a cytologically preneoplastic or premalignant form, hence changes in the composition of other biomolecules than genes can be assumed. Thus, alterations of the concentration of these biomolecules, including lipids, metabolites and proteins in tumor cells and margins, may be used for further diagnosis and characterization of tumor margins.

## Current state of tumor diagnostics

Currently, most imaging diagnostic tools such as magnetic resonance imaging (MRI), (micro-) computed tomography (μCT) or ultrasound, which provide additional information about tumor size, shape and microenvironment, are used to assist the surgeon in localizing the tumor pre- or post-operatively [16].

Until now, intraoperative diagnostics has been performed by specially trained pathologists using light microscopy for analysis of frozen sections looking for morphological changes associated with cancer. Determining the tumor margins by histological imaging has become the gold standard since the invention of the intraoperative frozen section, first published in 1905 (reviewed in [17]). Although universally applied, histological imaging has some major drawbacks: it is time-consuming [18], thus extending the time of anesthesia for the patient, it can be inaccurate due to technical limitations [19], it may be open to subjective interpretation [20–22], and only a certain number of sampling points are feasible.

Concerning time consumption, the transfer of a biopsy at room temperature to the pathologist is a factor that should be taken into consideration. After the transfer, prior to the sectioning, several inevitable factors can lead to the creation of frozen artifacts [23]. These frozen section artifacts result in a decrease of resolution of the microscopic images and, therefore, it is important to recognize these artifacts to obtain a correct interpretation [23,24].

In addition, potential biomarkers can be converted by enzymatic reactions [25,26], thus reducing the probability for using them as diagnostic markers. The overall time from resection until microscopic diagnosis amounts is 20–30 min; the tumor tissue is cut near the margin at first to save healthy tissue, so that the time of anesthesia is even more prolonged if a second or third analysis is requested [18]. Prepared under time pressure, if a patient is laying on the operation table [27], possible technical problems during slide-preparation are inevitable [8,28]. These technical challenges may lead to a variation in histopathologic diagnoses from the observer point of view. A study by van den Brekel *et al*. highlighted the subjectivity of human evaluation in histological images. Focusing on head and neck tumors, which are likely to metastasize to adjacent lymph nodes, significant inter- (K = 0.14–0.75) and intra-observer (K = 0.49–0.95) differences in terms of pathological diagnoses in fresh frozen tissue have been described [20]. An important prognostic factor for malignancy, the extranodal spread of tumor cells, was determined. Therefore, lymph nodes were evaluated by ten different pathologists, instructed to evaluate upon their own criteria. Histologically diagnosed cancer-free fields, adjacent to a tumor, can show functional characteristics of malignant cancer cells due to various reasons. Therefore, additional and alternative diagnostic methods that analyze tumor tissue in a faster and more accurate way than histology should be encouraged. With mass spectrometric methods designed for the operation room, factors such as mechanical damage during slide preparation and microscopically visible artefacts would not affect the result, since the analysis is based on objective biochemical characteristics such as the composition of lipids or proteins, not on morphology alone. These mentioned changes of the molecular phenotype are yet to be taken into account for routine diagnostics, but making use of it could lead to more efficient and personalized therapies in the future.

## MS-based methods for intraoperative tumor diagnostics

MS has been implemented as a diagnostic tool, being especially used in drug monitoring [29], newborn screening [30] and in the diagnosis of metabolic diseases [31]. In clinical research, the focus of mass spectrometric analysis has been on biomarker discovery, including proteomics, lipidomics and metabolomics [32,33].

Among the variety of molecules detectable with MS, metabolites, proteins and lipids have been used so far to provide useful information to distinguish between cancerous and healthy tissue.

To successfully identify a molecule as a qualified biomarker, the compound should be discriminable from other molecules, have a low limit of detection and should be interference-proof. Ideally the sample is easy, fast and fresh to obtain, has a high sensitivity (e.g. ≥ 0.9), specificity (e.g. ≥ 0.9) [34,35] and is a measurement for diagnosis and prognosis. In general, different classes of molecules could serve the purpose of a biomarker, due to the imbalance of tumor suppressing and promoting factors in cancer cells, controlling genetic alterations and therefore change of the composition of lipids, metabolites and proteins. Proteins, for example, participate in all steps of carcinogenesis; their concentrations, post-translational modifications and 3D structures are fairly unpredictable from genomic information. One of the biggest challenges to circumvent in the analysis of proteins is the long and complex sample preparation or chromatographic separation steps, to define a rapid valuable method for the operation theatre [36,37]. Without chromatographic separation, due to the superior ionization and desorption properties of lipids, protein signals are suppressed. Lipids – as potential biomarkers – are crucial for cellular membranes and are involved in cellular processes such as apoptosis [38], energy homeostasis and regulation of the molecular machinery [39,40] that determines size and replication in proliferating cells [41], providing information about cancerous growth. Due to the simpler sample preparation steps, lipids have proven to be detected reliably. In particular, fatty acids and phospholipids have been used as the discriminating molecules to distinguish between cancerous and healthy tissue. All techniques presented in this review [42–55], concentrate on applications using lipid analysis fitted for clinical application, as shown in Table 1. Based on a variety of sampling methods, the applications differ in their invasiveness (regarding the amount of tissue being removed), turnover time, cross contamination, preanalytical issues, surface scanning and cutting ability toward clinical use.

**Table 1.** **Overview about publications analyzing cancer specimen with mass spectrometric tools coupled to intraoperative sampling mechanisms.**

**Method**	**Tissue/Organ**	**Sample type**	**Statistical analysis**	**Reference**	**Year**
DESI	Xenografts from FaDu cells (SCC)	Frozen tissue, margins and cell culture	PCA	Woolman *et al*. [42]	2017

DESI	Pancreatic cancer tissue margins	Frozen tissue	LASSO	Eberlin *et al*. [43]	2016

DESI	Breast cancer tissue margins	Frozen tissue	PCA	Calligaris *et al*. [44]	2014

DESI	Glioma	Tissue smears	PCA, LDA	Pirro et al. [45]	2017

DESI	Epithelial ovarian carcinoma	Frozen tissue	PCA	Dória et al. [46]	2016

DESI	Gastric cancer and associated lymph node metastases	Frozen tissue	RMMC discriminant analysis, PCA	Abassi-Ghadi *et al*. [47]	2014

DESI	Breast cancer necrosis and viable regions	Frozen tissue, tissue smears	PCA, NMF	Tata *et al*. [48]	2016

DESI	Surgical glioma patients	Tissue smears	PCA, LDA	Pirro *et al*. [56]	2017

iKnife, REIMS	Colorectal cancer and colonic adenoma	*In vivo* patient material (polypectomy snare)	LDA	Alexander *et al*. [49]	2017

iKnife, REIMS	Breast cancer	*In vivo* and *ex vivo* (fresh and fresh frozen tissue, aspirated with monopolar handpiece)	PCA, LDA	St John *et al*. [50]	2016

iKnife, REIMS	Gastric, colorectal, liver, breast, lung and brain cancer; healthy and cancerous tissue	*In vivo* and *ex vivo* (<10 sec after excision)	PCA, LDA	Balog *et al*. [51]	2013

iKnife, REIMS	Colon adenocarcinoma and adematous polyps	*Ex vivo* biopsy (polypectomy snare)	PCA	Balog *et al*. [52]	2015

MasSpec Pen	Human: breast, lung, thyroid and ovary cancer Mouse: BT474 HER2+ breast cancer cells	Human: *Ex vivo*, fresh tissue (< 10 sec after excision). Mouse: *in vivo*	LASSO, (Leave-one-patient-out-crossvalidation), PCA	Zhang *et al*. [53]	2017

PIRL and DESI	Necrotic and viable LM2-4 human breast cancer xenografts	Frozen tissue	PLS-DA	Woolman *et al*. [54]	2017

PIRL	Medulloblastoma xenografts (six cell lines, two subgroups)	Frozen tissue, tissue smears	PLS-DA	Woolman *et al*. [55]	2017

DESI: Desorption electrospray ionization; PCA: Principal component analysis; LASSO: Least absolute shrinkage and selection operator; LDA: Linear discriminant analysis; PIRL: Picosecond infrared laser; REIMS: Rapid evaporative ionization mass spectrometry; RMMC: Recursive maximum margin criterion analysis; NMF: Non-negative matrix factorization; PLS-DA: Partial least squares discriminant analysis.

### DESI

**Figure 1.**
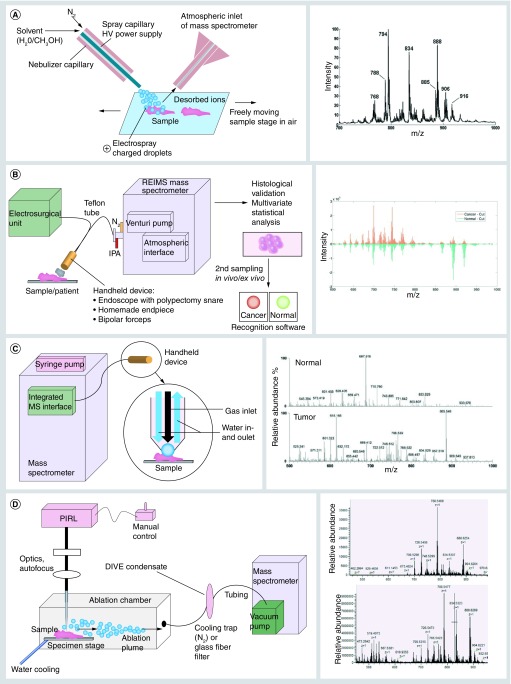
**Schematic figure of mass spectrometry based methods for intraoperative cancer diagnosis (left) with corresponding examples of mass spectra (right).** **(A)** Desorption electrospray ionization. Left: Schematic representation of desorption electrospray ionization. Under the influence of high voltage a methanol-water solution is sprayed on the sample surface, dissolving desorbed ions to be transferred in the atmospheric inlet of the mass spectrometer. Parameters like voltage, gas and liquid flow rate can be set. Right: Average lipid profile spectrum for all pixels images used for chemical prediction. Reproduced with permission from [45]. **(B)** iKnife. Left: Schematic illustration of rapid evaporative mass spectrometry coupled to eligible surgical devices. Surgical ion source and ion transfer setups for rapid evaporative mass spectrometry experiments are demonstrated using an endoscope, monopolar electrosurgery or commercially available bipolar electrosurgery. The aerosol is aspirated by an air jet pump through a teflon tube with a maximum of 3 m length. By histological validation and Principal Component Analysis, in a second run the recognition software differentiates cancer and normal tissue by comparing signal intensities in the recorded mass spectra. Most of the signals in the spectra represent lipid ions. Right: Mean spectral intensity for cancer and normal tissues during cutting. Reproduced with permission from [50]. **(C)** MasSpec pen. Left: A handheld device (MasSpec pen) is positioned on the sample surface and through the inlet channel a water droplet is exposed for 1 s to extract molecules from the tissue surface. Then, the channel is closed and after 2 s, gas inlet is opened to transport the water droplet (volume controlled by a syringe pump) via polytetrafluorethylen (PTFE) tubing driven by vacuum into the mass spectrometer. The system is triggered by a foot pedal connected to an integrated mass spectrometer inlet. Right: Representative negative ion mode mass spectra show distinct molecular profiles from normal (average of n = 3 mass spectra) and tumor (average of n = 3 mass spectra) tissues [53]. **(D)** Picosecond infrared laser. Left: Current instrument for sampling of tissue with picosecond infrared laser. For cryopreserved samples, the specimen stage is cooled down to -10°C, preventing thawing during laser irradiation. The ablation chamber is sealed to capture the aerosol in its complete volume, achieved by its architecture generating a laminar flow. The aerosol is trapped through PTFE-tubing as a frozen (cooling trap) or dry (glass fiber filter) condensate within seconds. Instead of the free laser beam a fiber can be utilized to adjust and operate flexibly. Right: Negative ion mode mass spectrum from picosecond infrared laser condensate of porcine thalamus (upper) and cerebral cortex (lower) directly infused into the MS without sample preparation. HV: High voltage; IPA: Isopropyl alcohol; PIRL: Picosecond infrared laser.

DESI was introduced in 2004 by the group of Graham Cooks [57], combining electrospray ionization with desorption ionization under atmospheric conditions. In DESI, a jet of gas and charged microdroplets is created using a standard pneumatic ESI sprayer. The jet of gas and charged microdroplets is directed onto a sample surface at angle α that is typically close to 45°. Analytes are ionized and desorbed from the surface by the charged microdroplets as a result of electrostatic and pneumatic forces [57]. The gas-phase ions of the desorbed analytes are subsequently transferred into the MS via an atmospheric pressure ion-transfer line or an extension tube that is mounted in front of the orifice of the MS. The ion-transfer line or extension tube further enhances the desorption and ionization process of the analytes [57,58] and guides the ions into the MS where the mass-to-charge ratios (m/z) of molecular ions and their abundances are measured (Figure 1). By choosing appropriate solvents, DESI is a nondestructive analysis technique, leaving the area of tissue intact In their 2011 work, Eberlin *et al*. demonstrated that the use of *N,N*-dimethylformamide-containing solvents as compared with acetonitrile and methanol-containing solvents preserves tissue integrity during DESI analysis [59]. The gentle ionization process transfers analytes such as lipids and proteins intact into the gas phase [60]. The chemistry of this desorption process can be compared with a solvent extraction experiment [61]. Depending on the solvent use, different molecules can be analyzed by DESI such as small molecules [62,63] and lipids [64–67] including cholesterol [68]. Hsu *et al*. detected intact proteins with masses up to 15 kDa with only minimum sample preparation from tissues by using nanospray desorption electrospray ionization (nanoDESI) [69], and recently the Eberlin group successfully combined DESI with field asymmetric waveform ion mobility (FAIMS; DESI-FAIMS-MS) to image proteins from mouse kidney, mouse brain and human ovarian and breast tissue samples [70]. The use of FAIMS increases signal-to-noise of protein ions and thus improves imaging contrast and quality. They further demonstrated MS-based on-tissue protein identification using abundant proteins, which represents an initial step toward in-depth tissue proteomics applications.

DESI is the most extensively used ambient ionization technique for MS imaging (MSI) and MS-based tissue diagnosis. DESI-MS is an interesting tool which provides a high potential for MS-based cancer diagnostics. Beneficial for surgical navigation, DESI can be used as an imaging technique. The architecture of the tissue sections used for the DESI-MS analysis remains largely intact and can subsequently be used for histopathological analysis by H&E staining [43].

Another electrospray based ambient ionization technique, called probe electrospray ionization, that uses a solid needle as a sampling probe, has been used for molecular diagnosis of malignant tumors based on imaging phospholipids and triacylglycerols [71] with high resolutions (0.1–0.5 mm). To ensure optimal conditions, this method allows for the adjustment of various parameters like the solvent concentration and flow rate, the surface angle α, the tip-to-surface distance and the voltage applied to the primary capillary [71,72]. Thus, optimal conditions for different tasks and analytes of interest like lipids or proteins can be established [57,60,71,73]. Clinically, DESI, as the most extensively used ambient ionization technique so far, has been successfully tested on resected tissue, frozen sections and fresh tissue smears. In addition to the variety of sample preparation, a broad amount of different cancerous organs have been analyzed; glandular tissue [48], skin [74], nervous tissue [56,75] and immunological tissue [47,76].

Tata *et al*. reported breast cancer viable and necrotic tissue by characterizing the presence of a ceramide ion (m/z 572.48 [Cer(d34:1) + Cl]^−^) from the viable cancer subregions; the absence of the ion of m/z 391.25 which is present in small abundance only in viable cancer subregions; and a slight increase in the relative intensity of known breast cancer biomarker fatty acid ions (m/z 281.25 [FA(18:1)-H]^−^ and 303.23 [FA(20:4)-H]^−^) [48]. Focusing on the spatial lipid distribution from tumor adjacent tissue in work by Calligaris *et al*., a mastectomy breast cancer margin study was established [44]. Patient samples (n = 14) from tumor center, margin, 2, and 5 cm away from the tumor were collected. Fatty acids like oleic acid were more abundant in the cancerous than in the healthy tissues evaluated by PCA.

Dória *et al*. focused on differentiation between serous, endometroid and clear cell carcinoma histotype with a rate >84% (histopathologic routine diagnosis accuracy is around 90%) [46]. Classifying subtypes of epithelial ovarian cancer by DESI-MSI, one of the most consistent changes in lipid composition is the PA (phosphatidic acid) class, with most significant higher peaks in the tumor tissue and its associated stroma compared with both controls of healthy ovary.

A successful attempt to distinguish cancerous samples and their matching lymph node metastases have been shown by Abbassi-Ghadi *et al*. in 2014 [47]. Gastric cancerous samples were analyzed compared with their associated lymph nodes by DESI matched with immunostain-histology. In order to obtain the Specific Molecular Ion Patterns ‘MISP’ to image and differentiate tumorous, metastatic and healthy areas, spectral profiles were subjected to recursive maximum margin criterion ‘RMMC’ discriminant analysis, for automated classification. Resulting in a higher abundance of phosphatidylethanolamines and phosphatidylinositol in the metastases than in the primary tumor, this method would objectify the process of lymph node metastasis identification. The authors also stated that by system optimization a timeframe of 10–20 min for data acquisition and a resolution of 200 μm – sufficient to detect micrometastases – could be achieved.

A detection of microscopic skin lesions by DESI-MS has been performed by Margulis *et al*., showing the differences in cancerous lesions and healthy tissue of basal cell carcinoma from Mohs micrographic surgery [74]. In this study, a resolution of 200 μm could be achieved to categorize each pixel as cancerous or healthy, based on 24 molecular ion signals with an overall diagnostic accuracy of 94.1%.

Focusing on gliomas and glioblastomas from human brain tissue samples, in three different projects marker-specific lipid profiles were analyzed, using DESI-MS [56,75,77–79]. Santagata *et al*. investigated frozen sections toward the prognostic lipid marker isocitrate dehydrogenase (IDH) via the tumor metabolite 2-hydroxyglutarate [77]. Based on this study, Jarmusch *et al*. compared frozen sections to corresponding tissue smears, resulting in the same chemical information given as in tissue sections [79]. Moreover, similar samples were also used by Jarmusch *et al*. to assess the difference between positive and negative ion mode generated results, revealing non-significant differences toward distinguishing between cancerous and noncancerous tissue in glioma [78]. Furthermore, Yannell *et al*. studied the mutation of IDH in 28 human glioma samples to prove that IDH is a powerful diagnostic factor in malignant gliomas [75]. Due to this groundwork, Pirro *et al*. investigated intraoperative biopsies from ten glioma patients, providing tissue smears, resulting in a sensitivity of 93% and a specificity of 83% in distinguishing high tumor cell percentage areas from healthy tissue by assessing the IDH mutation status within a 3-min timeframe from sample collection to result [56]. By performing the analysis using smear specimens instead of frozen sections, the time from sampling to diagnosis has been reduced significantly.

Taken together, DESI-MSI is an effective technology for rapid mapping of lipid distributions on cell membranes, showing excellent histological specificity and tissue classification. With the advantage of little to no sample preparation [56,80], enabling the correlation of lipid distribution in two or three dimensions [81], differentiating between cancerous, metastatic and healthy tissue [47,56,77,79], between different subtypes of cancer [46,82], and also between viable and necrotic tissue [48], DESI-MSI has a high potential in serving as an intraoperative real-time diagnostic tool in the future.

### REIMS – iKnife

In 2009 the group of Zoltan Takáts developed the ambient ionization technique rapid evaporative MS (REIMS) [83]. In the initial REIMS set-up Schaefer *et al*. used a commercially available handheld electrosurgical device equipped with an additional fluid line. The fluid line was used to aspirate the aerosol formed during tissue ablation. The aerosol was directly transferred with the help of a venturi pump into a MS. Similar to DESI, the REIMS spectra showed mainly different glycerophospholipids. However, in addition the intact lipids and their degradation products were also observed in REIMS spectra. The REIMS set-up has been optimized over the years to reduce contamination and to facilitate an efficient transfer of the aerosol over a distance of 1–2 meters from the surgical site to the MS [51,52,84]. It was discovered that in REIMS, formation of analyte ions detected in MS analysis takes place in the atmospheric interface of the MS via a droplet–surface collision phenomenon [85]. This resulted in a further refinement by implementing a heated collision surface in the atmospheric pressure interface of the MS. This heated collision surface additionally stops larger particles from entering the mass spectrometer that are generated during electrosurgical tissue ablation, due to high thermal energies leading to temperatures in the range of 700°C, the surface of the tissue resulting in tissue carbonization and smoke formation. For applications in the operating theatre, an electrosurgical knife (monopolar or bipolar diathermy knife) is equipped with an evacuation line connected to a mass spectrometer to transfer the aerosol over a distance of 2–3 m. With a spot size limited by handheld operation, a resolution of 0.5–2 mm is feasible [84]. During tissue dissection, the aerosol is aspirated, transferred to the MS and analyzed. The MS-derived lipid profiles are matched against a database of histologically annotated reference spectra and subjected to a multivariate statistical analysis [51]. The overall time for aerosol transfer, MS analysis and data classification takes approximately 0.5–2 s [50] and gives the surgeon real-time feedback if the dissected tissue is healthy or cancerous. The surgical application of the REIMS technology is known as the ‘intelligent knife – iKnife’ [49–51].

The REIMS – iKnife technology was applied in 2013, a large patient study by Balog *et al*. to diagnose tumor margins from different types of cancer was conducted [51]. For database generation, solid tumors and adjacent tissue of 302 *ex vivo* patient frozen samples were analyzed, collecting multiple data points by every 1 cm further from the visible tumor margin, to evaluate the lipidomic state through PCA and LDA statistical analysis. The analysis of human brain tissue for astrocytoma and metastatic tissue (84 patients with 12 different types of tumors), the real-time intraoperative interpretation of the results (in reference to histopathology) reached a sensitivity of 97.7% and a specificity of 96.5% in distinguishing between tumor and metastasis.

In 2016, Takáts *et al*. published an *ex vivo* colorectal cancer patient study using the iKnife [49]. Of the 28 recruited patients, the accuracy rate of the distinction between cancer and normal adjacent tissue amounted up to 90.5%, while the rate of distinction between cancer and adenoma was 94.4%. It was even possible to differentiate between the forms of histological subtypes, mucinous adenocarcinoma and regular adenocarcinoma with an AUC of 0.96. The colorectal cancer areas showed a high intensity for phosphatidylserines and bacterial phosphatidylglycerols, whereas the healthy tissue had high intensities for plasmalogens and triacylglycerols, and last the adenoma tissue demonstrated an overexpression of ceramides. However, concerning the human *in vivo* experiments, which were also described in this paper, the results were not provided to the clinical team during operation of the five different patients undergoing polypectomy. There was also no statistical analysis performed due to the small sample volume.

Later on, REIMS was also tested on a large number of fresh and frozen breast specimens (n = 249), tumorous and healthy samples were differentiated with a sensitivity of 90.9% and a specifcity of 98.8%.

Furthermore, an intraoperative proof-of-principle study was run to determine whether the iKnife *ex vivo* setup is applicable for *in vivo* surgery, by aspirating the electrosurgical aerosol produced sample model [50]. In these six case studies (n = 5462), 99.27% of the intraoperative spectra were interpretable by the *ex vivo* generated database.

Tested on patient tissue from three different hospitals, several different types of tumor samples have been analyzed with the iKnife and REIMS system resulting in a spectral reference library [49–51]. The iKnife is, among the described methods, the only application which has been applied *in vivo* on human cancer patients, resulting in real-time diagnosis sample, since the introduction of REIMS in 2009 [50,83]. Nearly 10 years of development work has resulted in the iKnife becoming one of the most advanced techniques, approaching real-time diagnostics for the operating room.

### MasSpec pen

Recently, the group of Livia S Eberlin described the development and application of an automated, biocompatible, disposable handheld device, the ‘MasSpec Pen’, for direct, real-time nondestructive sampling and molecular diagnosis of tissues by ambient ionization MS [53]. The MasSpec Pen has been optimized to minimize tissue damage and maximize both tissue-analyte extraction and solvent transfer to the MS device. The optimized handheld pen-sized probe consists of a syringe pump to deliver a defined water droplet to the sampling probe with a volume of 4–10 μl to dissolve and extract the tissue surface molecules. The sample volume is determined by the diameter of the reservoir at the probe tip, which is 1.5–5 mm diameter. The single water droplet is retained and exposed to the tissue surface for 3 s to allow an efficient analyte extraction. The extraction process of the MasSpec Pen is similar to liquid extraction surface analysis [86], liquid microjunction surface sampling probe [87] and nanoDESI [88]. Compared with liquid microjunction surface sampling probe and nanoDESI, the MasSpec Pen uses a single solvent drop instead of a continuous flow. In the initial set-up, the handheld pen is directly connected to a heated metal transfer tube of a high resolution quadrupole orbitrap MS via a PTFE tube (1.5 m length, inner diameter of 800 μm) [53]. The movement of the droplet from the reservoir to the MS was driven by the negative pressure of the MS vacuum system and could be controlled by a pump and two-way pinch valves. Vaporization and ionization of the extracted analytes occurs in the inlet region of the mass spectrometer. This process is similar to the described process of solvent assisted inlet ionization [89]. The mass spectra obtained with the MasSpec pen were similar to the mass spectra obtained with DESI including metabolites, lipids and proteins [53]. The entire analysis process of analyte extraction, transfer, MS analysis and tissue classification between healthy and unhealthy tissue takes 10 s and is supposed to be further reduced in the future.

In their initial publication Zhang *et al*. used the MasSpec Pen for *ex vivo* molecular analysis of 20 human cancer thin tissue sections and 253 human patient tissue samples [53]. The patient tissue samples included normal and cancerous tissues from four different organs (breast, lung, thyroid and ovary). Zhang *et al*. generated a histologically validated database containing mass spectra of rich molecular profiles that were characterized by a variety of potential cancer biomarkers identified as metabolites, lipids and proteins. Using statistical classifiers built from this database, Zhang *et al*. were able to diagnose cancerous and noncancerous tissue with a high sensitivity (96.4%), specificity (96.2%) and overall accuracy (96.3%). They were also able to predict benign and malignant thyroid tumors and different histologic subtypes of lung cancer. Notably, the MasSpec pen allowed accurate diagnosis of cancer in marginal tumor regions presenting mixed histologic composition and accurate *in vivo* cancer diagnosis during surgery performed in tumor-bearing mouse models [53].

Since no labeling occurs, the exact margins are not visualized and the exact resection might still be challenging clinically. Sample carry-over effects in case of endoscopic surgeries in abdominal or chest area could also present an obstacle: discarding the tip is not an option, therefore, the MasSpec Pen might represent a potential source for cross contamination. For this special purpose, biocompatible, autoclavable materials like polylactid-co-glycolid, a common suture material, also used for degradable implants, could be the solution [90].

In the described experiments, working with molecules dissolved from the tissue surface by a droplet of water and an exposure time of 3 s, the MasSpec Pen represents a noninvasive technique in tumor diagnostics.

### PIRL

Lasers, technically capable of cutting tissue at a single cell level, have always been a concept of interest for surgical procedures. The first CO_2_-infrared lasers have been used for the excision of sensitive tissue areas with the aim to irradiate a broad variety of tissue materials with different density. Mainly applied for surgery in the 1990s [91], the CO_2_-laser was later integrated in mass spectrometric measurements (double focusing mass spectrometer with photoplate detection) by laser desorption ionization [92,93].

In 2009, the group of Dwayne Miller introduced a new concept in laser surgery based on a PIRL [94]. PIRL is specifically tuned to the strong OH vibration stretching band in water molecules to drive ablation processes faster than nucleation growth or acoustic energy transfer to the adjacent tissue [95]. The energy absorbed by the tissue-own water molecules is completely converted into translational degrees of freedom rather than being lost to surrounding tissue through thermal or acoustic transport. This process called ‘desorption by impulsive vibrational excitation’ [96] drives the water molecules into the gas phase without thermal or shock-wave damage to the ambient tissue, resulting in cold tissue vaporization [97]. The unique properties of PIRL enable precise and accurate ablation with minimal cellular damages to the surrounding tissue, significantly reducing scar formation compared with millisecond infrared lasers commonly used in medical laser surgery [97–104]. Next to the medical application, the group of Hartmut Schlüter showed that PIRL ablation released proteins intact from tissues without changing their exact chemical composition and that posttranslational modifications and enzyme activities of the PIRL-ablated proteins remained unaltered [96]. In another study Kwiatkowski *et al*. it was shown that due to the ultrafast transfer of proteins from tissues via gas phase into frozen condensates of the aerosols, intact protein species were exposed to a lesser extent to enzymatic degradation reactions using PIRL ablation compared with conventional homogenization and protein extraction methods [105]. Thus, cold tissue vaporization by PIRL gives a unique access to the *in vivo* protein species/proteoform composition [106] of tissues providing a snapshot of the *in vivo* proteome composition. The gentle process of PIRL ablation was confirmed by a study of Ren *et al*. in which they showed that protein conformations are not significantly disturbed and even large macromolecular structures such as viruses are extracted intact without losing their biological activity [107]. They further showed that biological samples can be taken down to the level of individual cells using PIRL offering a finer resolution than the finest biopsy needles, thus having the potential to enable minimally invasive biopsies.

Recently, the group of Arash Zarrine-Afsar developed a handheld PIRL probe and MS interface [55]. They used a 2 m long tygon tube (1.6 mm inner diameter) that was connected to the ion transfer tube of a commercial DESI source (Waters). The temperature of the ion transfer capillary and MS source was sufficient to dissolve phospholipids and fatty acids that were released from the tissue with the handheld PIRL probe. Using the handheld PIRL probe tissue-specific MS profiles were obtained within 5–10 s after tissue ablation, thus enabling real-time analysis of the ablation plume. In their study Woolman *et al*. examined breast cancer tissue slices with PIRL coupled to polarimetric imaging [54]. LM2-4 human breast cancer xenografts from mice were cryopreserved and ablated 10 s with PIRL to be scanned for viable and necrotic tissue areas obtaining real time spectra showing mainly phospholipids and fatty acids. The authors were able to classify and differentiate necrotic cancer sites from viable cancers sites in the tissue based on specific molecular profiles using multivariate statistical methods. In another study, Woolman *et al*. applied the handheld PIRL probe for *ex-vivo* differentiation and classification different medulloblastoma subtypes [55]. The PIRL-MS analysis offered a 98% success rate in subgroup determination observed over 194 tumor tissues collected from 19 independent tumors. The classification and differentiation was based on MS profiles containing a variety of fatty acids, glycerophosphates, glycerophosphoglycerols and glycerophosphocholines applying multivariate statistical methods. In addition, Woolman *et al*. collected PIRL ablated tissue material on a filter paper placed in vacuum line of a suction pump unit. The lipids were extracted from the filter and subjected to high-resolution liquid chromatography–MS (LC–MS) analysis to identify the lipid species that contributed most to the statistical discrimination between the different medulloblastoma subtypes [55].

## Conclusion & future perspective

In the last decade, the field of intraoperative mass spectrometric tools for cancer resection and diagnostics has gained increased attention. Existing surgical tools have been connected to mass spectrometers for real-time on-line analysis of cancer tissues. Sample pick-up, sample transfer as well as ambient desorption and ionization were optimized as a groundbreaking step, allowing immediate analysis of tissues for investigating if the cancer tissue has been completely removed.

From this development, two categories of tools have emerged: sampling and cutting tools, which are able to resect and diagnose tissue intraoperatively; and tools providing a diagnosis exclusively, as shown in Table 2 [50,53,55,108]. The first category includes the electrocautery knife coupled to a mass spectrometer, termed iKnife, that provides real-time information about the tissue while resecting, thereby giving the surgeon an orientation with respect to the resection margins. The PIRL scalpel coupled to a mass spectrometer is comparable to the iKnife regarding cutting and detecting tissue molecules in real time. In contrast to the iKnife, the PIRL scalpel has the advantage that the cells adjacent to the cut will not be damaged. In addition, because PIRL has a fast scanning rate, PIRL-based systems are a promising intraoperative diagnostic tool for screening surfaces of tissues for the absence of tumor cells. However, the current analysis time of 5–10 s per spot with a spot size of 200 μm for sampling and data analysis [55] is comparatively slow for diagnosis of large tissue surfaces in clinical practice. This timeframe is the limiting factor at this time and presents a challenge that must be overcome in the future for PIRL to be used for diagnostic tissue surface screening. With a resolution of 4 mm in blade width, the iKnife has a low resolution compared with the other methods discussed in this review. Thus, the iKnife might produce false negative results due to the dilution of tumor and healthy cells [49]. Since the iKnife massively damages the tissue adjacent to the cutting area due to very high temperatures, it is impossible to be certain by histological investigation of the resected tumor tissue of having fully removed cancer cells, because morphology is no longer visible in the carbonized area.

**Table 2.** **Overview of mass spectrometry-based methods for intraoperative cancer diagnosis.**

**Quality**	**Method**	**DESI**	**iKnife**	**MasSpec pen**	**DESI-PIRL**	**PIRL**
Speed		1 s per sampling point [108]	<2 s [50]	3 s per sampling point [53]	5–10 s per sampling point [55]	<5 s

Cross contamination		No	Yes	No	No	No

Invasiveness		Yes	Yes	No	Yes minimal	Yes minimal

Pre-analytical issues		Redox reactions can be assumed [109,110]	Heat degradation, carbonization	Unlikely	Not observed	Not observed

Surface scanning		No	No	Yes, discontinuously	Yes	Yes

Cutting abilities		No	Yes	No	No	Yes

Resolution		Electrospray: 200 μm [47,74]	Blade: 4 mm [50]	Probe tip: 1.5–5 mm diameter [53]	Fibre: 425 μm [54,55]	Fibre: 200–250 μm [104,111] Free beam: 250 μm [97]

Tissue damage		Low	High	Very low	Low	Low, minimal scar formation [103]

DESI: Desorption electrospray ionization; PIRL: Picosecond infrared laser.

The DESI and the MasSpec pen constitute a category of diagnostic tools with which surfaces of tissues can be analyzed without damaging the cells of the analyzed tissues. As an improvement or substitute for the analysis of frozen sections, DESI could be used outside the operation room for a rapid diagnosis. The MasSpec pen may be applied directly to the patient for intraoperative diagnostics. However, scanning of larger tissue surface to acquire real-time diagnosis is not yet feasible with this method. Analysis with the MasSpec pen only requires the application of a droplet of water onto the tissue surface without any pressure, therefore being noninvasive.

The MS-pen can achieve online classification of healthy and unhealthy tissue in less than 10 seconds [53]. Tissue scanning with PIRL has a comparable speed. PIRL is a minimally destructive method of tissue ablation [94–100], meaning that the cells adjacent to the ablated cells are not damaged, the ablated tissue of course is removed and thereby destroyed. This is not the case with the nondestructive MasSpec pen method [53], where the analyzed tissue is available for further diagnostic analysis such as histology. The PIRL-MS platform offers potential for future *in vivo* applications where therapy and diagnosis can be performed in parallel in form of a theragnostic tool. The device as a scalpel can be positioned within the operating room and used as a surgical tool, providing a way to objectify decisions for the surgeon. However, future experiments are mandatory to prove that PIRL-MS can be applied to *in vivo* diagnostics, similar to the MS pen [53].

All diagnostic tools based on ambient ionization techniques described above allow the detection of tissue molecules with minimal sample preparation. Also, one essential requirement for application of all of these methods for intraoperative diagnostics for discriminating cancer tissue from healthy tissue is the knowledge of biomarkers, which can be patterns of signals of lipids. By identifying such biomarkers, the generation of databases for each type of cancer will help to classify cancerous and healthy tissue. However, technological grounds have been set to make intraoperative diagnostics of tumors a realistic approach within the next few years.

**Executive summary**

- The need for exact tumor margin assessment in surgery of cancer tissue is high and therefore accelerating the development of sensitive diagnostic tools is required.
- Screening for biomarkers with mass spectrometry coupled to scanning devices provides sensitive and specific tools for intraoperative diagnosis. These tools are either an improvement of the frozen section process or can be used as a theragnostic cutting instrument simultaneously.
- Described methods vary in invasiveness, cross contamination risk, cutting abilities, speed, chemical reactions induced by the device, handling qualities, spatial resolution and clinical study status.

## References

[1] Bozzetti F, Bonfanti G, Bufalino R (1982). Adequacy of margins of resection in gastrectomy for cancer. *Ann. Surg.*.

[2] Shin D, Park SS (2013). Clinical importance and surgical decision-making regarding proximal resection margin for gastric cancer. *World J. Gastrointest. Oncol.*.

[3] Persing S, Jerome MA, James TA (2015). Surgical margin reporting in breast conserving surgery: does compliance with guidelines affect re-excision and mastectomy rates?. *Breast.*.

[4] Heiss N, Rousson V, Ifticene-Treboux A, Lehr HA, Delaloye JF (2017). Risk factors for positive resection margins of breast cancer tumorectomy specimen following breast-conserving surgery. *Horm. Mol. Biol. Clin. Investig.*.

[5] Slaughter DP, Southwick HW, Smejkal W (1953). Field cancerization in oral stratified squamous epithelium; clinical implications of multicentric origin. *Cancer*.

[6] Rubin H (2011). Fields and field cancerization: the preneoplastic origins of cancer: asymptomatic hyperplastic fields are precursors of neoplasia, and their progression to tumors can be tracked by saturation density in culture. *Bioessays*.

[7] Dakubo GD, Jakupciak JP, Birch-Machin MA, Parr RL (2007). Clinical implications and utility of field cancerization. *Cancer Cell Int.*.

[8] Jeevan R, Cromwell DA, Trivella M (2012). Reoperation rates after breast conserving surgery for breast cancer among women in England: retrospective study of hospital episode statistics. *BMJ (Clin. Res. Ed.)*.

[9] Jaiswal G, Jaiswal S, Kumar R, Sharma A (2013). Field cancerization: concept and clinical implications in head and neck squamous cell carcinoma. *J. Exp. Ther. Oncol.*.

[10] Braakhuis BJ, Tabor MP, Kummer JA, Leemans CR, Brakenhoff RH (2003). A genetic explanation of Slaughter's concept of field cancerization: evidence and clinical implications. *Cancer Res.*.

[11] Leemans CR, Braakhuis BJ, Brakenhoff RH (2011). The molecular biology of head and neck cancer. *Nat. Rev. Cancer*.

[12] Al-Ghnaniem R, Camprodon RA, Kocher HM (2008). Strategy to reduce the risk of positive pancreatic resection margin at pancreatico-duodenectomy. *ANZ J.. Surg.*.

[13] Tsao JL, Yatabe Y, Salovaara R (2000). Genetic reconstruction of individual colorectal tumor histories. *Proc. Natl Acad. Sci. USA*.

[14] Braathen LR, Morton CA, Basset-Seguin N (2012). Photodynamic therapy for skin field cancerization: an international consensus. International Society for Photodynamic Therapy in Dermatology. *J. Eur. Acad. Dermatol. Venereol.*.

[15] Zeki SS, Mcdonald SA, Graham TA (2011). Field cancerization in Barrett's esophagus. *Discov Med*.

[16] Sakr RA, Poulet B, Kaufman GJ, Nos C, Clough KB (2011). Clear margins for invasive lobular carcinoma: a surgical challenge. *Eur. J. Surg. Oncol.*.

[17] Gal AA, Cagle PT (2005). The 100-year anniversary of the description of the frozen section procedure. *JAMA*.

[18] Novis DA, Zarbo RJ (1997). Interinstitutional comparison of frozen section turnaround time. A College of American Pathologists Q-Probes study of 32868 frozen sections in 700 hospitals. *Arch. Pathol. Lab. Med.*.

[19] Jaafar H (2006). Intra-operative frozen section consultation: concepts, applications and limitations. *Malaysian J. Med. Sci. : MJMS*.

[20] Van Den Brekel MW, Lodder WL, Stel HV, Bloemena E, Leemans CR, Van Der Waal I (2012). Observer variation in the histopathologic assessment of extranodal tumor spread in lymph node metastases in the neck. *Head Neck*.

[21] Stel HV, Vroom TM, Blok P, Van Heerde P, Meijer CJ (1989). Therapy-relevant discrepancies between diagnoses of institutional pathologists and experienced hematopathologists in the diagnosis of malignant lymphoma. *Pathol. Res. Pract.*.

[22] Hopton DS, Thorogood J, Clayden AD, Mackinnon D (1989). Observer variation in histological grading of breast cancer. *Eur. J. Surg. Oncol.*.

[23] Desciak EB, Maloney ME (2000). Artifacts in frozen section preparation. *Dermatol. Surg.*.

[24] Ishii M, Bishop JA, Gallia GL (2017). Assessment of frozen section margin analysis during olfactory neuroblastoma surgery. *Laryngoscope*.

[25] Tworoger SS, Hankinson SE (2006). Collection, processing, and storage of biological samples in epidemiologic studies: sex hormones, carotenoids, inflammatory markers, and proteomics as examples. *Cancer Epidemiol. Biomark. Prevent.*.

[26] Rai AJ, Gelfand CA, Haywood BC (2005). HUPO Plasma Proteome Project specimen collection and handling: towards the standardization of parameters for plasma proteome samples. *Proteomics*.

[27] Horne GJ, Barber DF, Bruecks AK, Maung RT, Trotter MJ (2009). Workload measurement in subspecialty dermatopathology. *J. Clin. Pathol.*.

[28] Szaloki T, Toth V, Tiszlavicz L, Czako L (2006). Flat gastric polyps: results of forceps biopsy, endoscopic mucosal resection, and long-term follow-up. *Scand. J. Gastroenterol.*.

[29] Garg U, Zhang YV (2016). Mass spectrometry in clinical laboratory: applications in therapeutic drug monitoring and toxicology. *Methods Mol. Biol. (Clifton, N.J.)*.

[30] Ombrone D, Giocaliere E, Forni G, Malvagia S, La Marca G (2016). Expanded newborn screening by mass spectrometry: new tests, future perspectives. *Mass Spectrom. Rev.*.

[31] Leonard JV, Dezateux C (2002). Screening for inherited metabolic disease in newborn infants using tandem mass spectrometry: further assessment of performance and outcome is needed. *BMJ : Br. Med. J.*.

[32] Zhou W, Petricoin EF, Longo C (2017). Mass Spectrometry-Based Biomarker Discovery. *Methods Mol. Biol. (Clifton, N.J.)*.

[33] Steffen P, Kwiatkowski M, Robertson WD (2016). Protein species as diagnostic markers. *J. Proteomics*.

[34] Brower V (2011). Biomarkers: Portents of malignancy. *Nature*.

[35] Rosenling T, Stoop MP, Smolinska A (2011). The impact of delayed storage on the measured proteome and metabolome of human cerebrospinal fluid. *Clin. Chem.*.

[36] Wiseman JM, Puolitaival SM, Takats Z, Cooks RG, Caprioli RM (2005). Mass spectrometric profiling of intact biological tissue by using desorption electrospray ionization. *Angew Chem. Int. Ed. Engl.*.

[37] Todd PJ, Schaaff TG, Chaurand P, Caprioli RM (2001). Organic ion imaging of biological tissue with secondary ion mass spectrometry and matrix-assisted laser desorption/ionization. *J. Mass Spectrom.*.

[38] Carpinteiro A, Dumitru C, Schenck M, Gulbins E (2008). Ceramide-induced cell death in malignant cells. *Cancer Lett.*.

[39] Wymann MP, Schneiter R (2008). Lipid signalling in disease. *Nat. Rev. Mol. Cell Biol.*.

[40] Hannun YA, Obeid LM (2008). Principles of bioactive lipid signalling: lessons from sphingolipids. *Nat. Rev. Mol. Cell Biol.*.

[41] Yuan TL, Cantley LC (2008). PI3K pathway alterations in cancer: variations on a theme. *Oncogene*.

[42] Woolman M, Tata A, Dara D (2017). Rapid determination of the tumour stroma ratio in squamous cell carcinomas with desorption electrospray ionization mass spectrometry (DESI-MS): a proof-of-concept demonstration. *Analyst*.

[43] Eberlin LS, Margulis K, Planell-Mendez I (2016). Pancreatic cancer surgical resection margins: molecular assessment by mass spectrometry imaging. *PLoS Med.*.

[44] Calligaris D, Caragacianu D, Liu X (2014). Application of desorption electrospray ionization mass spectrometry imaging in breast cancer margin analysis. *Proc. Natl Acad. Sci. USA*.

[45] Pirro V, Jarmusch AK, Alfaro CM, Hattab EM, Cohen-Gadol AA, Cooks RG (2017). Utility of neurological smears for intrasurgical brain cancer diagnostics and tumour cell percentage by DESI-MS. *Analyst*.

[46] Doria ML, Mckenzie JS, Mroz A (2016). Epithelial ovarian carcinoma diagnosis by desorption electrospray ionization mass spectrometry imaging. *Sci. Rep.*.

[47] Abbassi-Ghadi N, Veselkov K, Kumar S (2014). Discrimination of lymph node metastases using desorption electrospray ionisation-mass spectrometry imaging. *Chem. Commun. (Camb.)*.

[48] Tata A, Woolman M, Ventura M (2016). Rapid detection of necrosis in breast cancer with desorption electrospray ionization mass spectrometry. *Sci. Rep.*.

[49] Alexander J, Gildea L, Balog J (2017). A novel methodology for in vivo endoscopic phenotyping of colorectal cancer based on real-time analysis of the mucosal lipidome: a prospective observational study of the iKnife. *Surg. Endosc.*.

[50] St John ER, Balog J, Mckenzie JS (2017). Rapid evaporative ionisation mass spectrometry of electrosurgical vapours for the identification of breast pathology: towards an intelligent knife for breast cancer surgery. *Breast Cancer Res.*.

[51] Balog J, Sasi-Szabo L, Kinross J (2013). Intraoperative tissue identification using rapid evaporative ionization mass spectrometry. *Sci. Transl. Med.*.

[52] Balog J, Kumar S, Alexander J (2015). In vivo endoscopic tissue identification by rapid evaporative ionization mass spectrometry (REIMS). *Angew. Chem. (Int. Ed. English)*.

[53] Zhang J, Rector J, Lin JQ (2017). Nondestructive tissue analysis for *ex vivo* and *in vivo* cancer diagnosis using a handheld mass spectrometry system. *Sci. Transl. Med.*.

[54] Woolman M, Gribble A, Bluemke E (2017). Optimized mass spectrometry analysis workflow with polarimetric guidance for *ex vivo* and *in situ* sampling of biological tissues. *Sci. Rep.*.

[55] Woolman M, Ferry I, Kuzan-Fischer CM (2017). Rapid determination of medulloblastoma subgroup affiliation with mass spectrometry using a handheld picosecond infrared laser desorption probe. *Chem. Sci.*.

[56] Pirro V, Alfaro CM, Jarmusch AK, Hattab EM, Cohen-Gadol AA, Cooks RG (2017). Intraoperative assessment of tumor margins during glioma resection by desorption electrospray ionization-mass spectrometry. *Proc. Natl Acad. Sci. USA*.

[57] Takats Z, Wiseman JM, Gologan B, Cooks RG (2004). Mass spectrometry sampling under ambient conditions with desorption electrospray ionization. *Science (New York, N.Y.)*.

[58] Cooks RG, Ouyang Z, Takats Z, Wiseman JM (2006). Detection technologies. ambient mass spectrometry. *Science*.

[59] Eberlin LS, Ferreira CR, Dill AL, Ifa DR, Cheng L, Cooks RG (2011). Nondestructive, histologically compatible tissue imaging by desorption electrospray ionization mass spectrometry. *Chembiochem*.

[60] Nefliu M, Smith JN, Venter A, Cooks RG (2008). Internal energy distributions in desorption electrospray ionization (DESI). *J. Am. Soc. Mass Spectrom.*.

[61] Badu-Tawiah AK, Eberlin LS, Ouyang Z, Cooks RG (2013). Chemical aspects of the extractive methods of ambient ionization mass spectrometry. *Annu. Rev. Phys. Chem.*.

[62] Jackson AU, Shum T, Sokol E, Dill A, Cooks RG (2011). Enhanced detection of olefins using ambient ionization mass spectrometry: Ag^+^ adducts of biologically relevant alkenes. *Anal. Bioanal. Chem.*.

[63] Badu-Tawiah A, Cooks RG (2010). Enhanced ion signals in desorption electrospray ionization using surfactant spray solutions. *J. Am. Soc. Mass. Spectrom.*.

[64] Manicke NE, Nefliu M, Wu C (2009). Imaging of lipids in atheroma by desorption electrospray ionization mass spectrometry. *Anal. Chem.*.

[65] Eberlin LS, Ferreira CR, Dill AL, Ifa DR, Cooks RG (2011). Desorption electrospray ionization mass spectrometry for lipid characterization and biological tissue imaging. *Biochim. Biophys. Acta*.

[66] Eberlin LS, Dill AL, Golby AJ (2010). Discrimination of human astrocytoma subtypes by lipid analysis using desorption electrospray ionization imaging mass spectrometry. *Angew. Chem. (Int. Ed. English)*.

[67] Gouw AM, Eberlin LS, Margulis K (2017). Oncogene KRAS activates fatty acid synthase, resulting in specific ERK and lipid signatures associated with lung adenocarcinoma. *Proc. Natl Acad. Sci. USA*.

[68] Wu C, Ifa DR, Manicke NE, Cooks RG (2009). Rapid, direct analysis of cholesterol by charge labeling in reactive desorption electrospray ionization. *Anal. Chem.*.

[69] Hsu CC, Chou PT, Zare RN (2015). Imaging of proteins in tissue samples using nanospray desorption electrospray ionization mass spectrometry. *Anal. Chem.*.

[70] Garza KY, Feider CL, Klein DR, Rosenberg JA, Brodbelt JS, Eberlin LS (2018). Desorption electrospray ionization mass spectrometry imaging of proteins directly from biological tissue sections. *Anal. Chem.*.

[71] Green FM, Stokes P, Hopley C, Seah MP, Gilmore IS, O'connor G (2009). Developing repeatable measurements for reliable analysis of molecules at surfaces using desorption electrospray ionization. *Anal. Chem.*.

[72] Bennet RV, Gamage CM, Fernandez FM (2013). Imaging of biological tissues by desorption electrospray ionization mass spectrometry. *J. Visual. Exp. : JoVE*.

[73] Nefliu M, Smith JN, Venter A, Cooks RG (2008). Internal energy distributions in desorption electrospray ionization (DESI). *J. Am. Soc. Mass Spectrom.*.

[74] Margulis K, Chiou AS, Aasi SZ, Tibshirani RJ, Tang JY, Zare RN (2018). Distinguishing malignant from benign microscopic skin lesions using desorption electrospray ionization mass spectrometry imaging. *Proc. Natl Acad. Sci. USA*.

[75] Yannell KE, Smith K, Alfaro CM, Jarmusch AK, Pirro V, Cooks RG (2017). N-acetylaspartate and 2-hydroxyglutarate assessed in human brain tissue by mass spectrometry as neuronal markers of oncogenesis. *Clinical chemistry*.

[76] Abbassi-Ghadi N, Golf O, Kumar S (2016). Imaging of esophageal lymph node metastases by desorption electrospray ionization mass spectrometry. *Cancer Res.*.

[77] Santagata S, Eberlin LS, Norton I (2014). Intraoperative mass spectrometry mapping of an onco-metabolite to guide brain tumor surgery. *Proc. Natl Acad. Sci. USA*.

[78] Jarmusch AK, Alfaro CM, Pirro V, Hattab EM, Cohen-Gadol AA, Cooks RG (2016). Differential lipid profiles of normal human brain matter and gliomas by positive and negative mode desorption electrospray ionization – mass spectrometry imaging. *PloS ONE*.

[79] Jarmusch AK, Pirro V, Baird Z, Hattab EM, Cohen-Gadol AA, Cooks RG (2016). Lipid and metabolite profiles of human brain tumors by desorption electrospray ionization-MS. *Proc. Natl Acad. Sci. USA*.

[80] Donnellan KA, Pitman KT, Cannon CR, Replogle WH, Simmons JD (2009). Intraoperative laryngeal nerve monitoring during thyroidectomy. *Arch. Otolaryngol. Head Neck Surg.*.

[81] Wiseman JM, Ifa DR, Song Q, Cooks RG (2006). Tissue imaging at atmospheric pressure using desorption electrospray ionization (DESI) mass spectrometry. *Angew. Chem. (Int. Ed. English)*.

[82] Eberlin LS, Norton I, Dill AL (2012). Classifying human brain tumors by lipid imaging with mass spectrometry. *Cancer Res.*.

[83] Schafer KC, Denes J, Albrecht K (2009). In vivo, in situ tissue analysis using rapid evaporative ionization mass spectrometry. *Angew. Chem. (Int. Ed. English)*.

[84] Balog J, Szaniszlo T, Schaefer KC (2010). Identification of biological tissues by rapid evaporative ionization mass spectrometry. *Anal. Chem.*.

[85] Balog J, Perenyi D, Guallar-Hoyas C (2016). Identification of the species of origin for meat products by rapid evaporative ionization mass spectrometry. *J. Agric. Food Chem.*.

[86] Kertesz V, Van Berkel GJ (2010). Fully automated liquid extraction-based surface sampling and ionization using a chip-based robotic nanoelectrospray platform. *J. Mass Spectrom. : JMS*.

[87] Kertesz V, Ford MJ, Van Berkel GJ (2005). Automation of a surface sampling probe/electrospray mass spectrometry system. *Anal. Chem.*.

[88] Laskin J, Heath BS, Roach PJ, Cazares L, Semmes OJ (2012). Tissue imaging using nanospray desorption electrospray ionization mass spectrometry. *Anal. Chem.*.

[89] Pagnotti VS, Chubatyi ND, Mcewen CN (2011). Solvent assisted inlet ionization: an ultrasensitive new liquid introduction ionization method for mass spectrometry. *Anal. Chem.*.

[90] Astete CE, Sabliov CM (2006). Synthesis and characterization of PLGA nanoparticles. *J. Biomater. Sci. Polym. Ed.*.

[91] Roberts TL, Lettieri JT, Ellis LB (1996). CO_2_ laser resurfacing: recognizing and minimizing complications. *Aesth. Surg. J.*.

[92] Toodle DE (1981). Mass spectrometry of the CO_2_ laser plasma.. *DTIC*.

[93] Posthumus MA, Kistemaker PG, Meuzelaar HLC, Ten Noever De Brauw MC (1978). Laser desorption-mass spectrometry of polar nonvolatile bio-organic molecules. *Anal. Chem.*.

[94] Franjic K, Cowan ML, Kraemer D, Miller RJ (2009). Laser selective cutting of biological tissues by impulsive heat deposition through ultrafast vibrational excitations. *Opt. Express*.

[95] Franjic K, Miller D (2010). Vibrationally excited ultrafast thermodynamic phase transitions at the water/air interface. *Phys. Chem. Chem. Phys. : PCCP*.

[96] Kwiatkowski M, Wurlitzer M, Omidi M (2015). Ultrafast extraction of proteins from tissues using desorption by impulsive vibrational excitation. *Angew. Chem. (Int. Ed. English)*.

[97] Bottcher A, Kucher S, Knecht R (2015). Reduction of thermocoagulative injury via use of a picosecond infrared laser (PIRL) in laryngeal tissues. *Eur. Arch. Otorhinolaryngol.*.

[98] Amini-Nik S, Kraemer D, Cowan ML (2010). Ultrafast mid-IR laser scalpel: protein signals of the fundamental limits to minimally invasive surgery. *PloS One*.

[99] Hess M, Hildebrandt MD, Muller F (2013). Picosecond infrared laser (PIRL): an ideal phonomicrosurgical laser?. *Eur. Arch. Otorhinolaryngol.*.

[100] Jowett N, Wollmer W, Mlynarek AM (2013). Heat generation during ablation of porcine skin with erbium: YAG laser vs a novel picosecond infrared laser. *JAMA Otolaryngol.*.

[101] Bottcher A, Clauditz TS, Knecht R (2013). A novel tool in laryngeal surgery: preliminary results of the picosecond infrared laser. *Laryngoscope*.

[102] Jowett N, Wollmer W, Reimer R (2014). Bone ablation without thermal or acoustic mechanical injury via a novel picosecond infrared laser (PIRL). *Otolaryngol. Head Neck Surg.*.

[103] Petersen H, Tavakoli F, Kruber S (2016). Comparative study of wound healing in rat skin following incision with a novel picosecond infrared laser (PIRL) and different surgical modalities. *Lasers Surg. Med.*.

[104] Petersen H, Gliese A, Stober Y (2018). Picosecond infrared laser (PIRL) application in stapes surgery – first experience in human temporal bones. *Otol. Neurotol.*.

[105] Kwiatkowski M, Wurlitzer M, Krutilin A (2016). Homogenization of tissues via picosecond-infrared laser (PIRL) ablation: giving a closer view on the *in-vivo* composition of protein species as compared to mechanical homogenization. *J. Proteomics*.

[106] Aebersold R, Agar JN, Amster IJ (2018). How many human proteoforms are there?. *Nat. Chem. Biol.*.

[107] Ren L, Robertson WD, Reimer R (2015). Towards instantaneous cellular level bio diagnosis: laser extraction and imaging of biological entities with conserved integrity and activity. *Nanotechnology*.

[108] Tillner J, Wu V, Jones EA (2017). Faster, more reproducible DESI-MS for biological tissue imaging. *J. Am. Soc. Mass Spectrom.*.

[109] Jurva U, Wikstrom HV, Weidolf L, Bruins AP (2003). Comparison between electrochemistry/mass spectrometry and cytochrome p450-catalyzed oxidation reactions. *Rapid Commun. Mass Spectrom. : RCM*.

[110] Liu P, Lanekoff IT, Laskin J, Dewald HD, Chen H (2012). Study of electrochemical reactions using nanospray desorption electrospray ionization mass spectrometry. *Anal. Chem.*.

[111] Mehlan J, Uschold S, Hansen NO (2018). Picosecond infrared laser fiber-assisted sclerostomy (PIRL-FAST): a first proof of principle analysis. *Ophthalmologe*.

